# Gastrointestinal Emergencies in Neonates: A Review Article

**DOI:** 10.7759/cureus.30538

**Published:** 2022-10-21

**Authors:** Shivani Akre, Kapil Sharma, Swarupa Chakole, Mayur B Wanjari

**Affiliations:** 1 Department of Medicine, Jawaharlal Nehru Medical College, Datta Meghe Institute of Medical Sciences, Wardha, IND; 2 Department of Community Medicine, Jawaharlal Nehru Medical College, Datta Meghe Institute of Medical Sciences, Wardha, IND; 3 Department of Research, Jawaharlal Nehru Medical College, Datta Meghe Institute of Medical Sciences, Wardha, IND

**Keywords:** life-threatening, embryonic, low birth weight, perforation, neonate

## Abstract

Numerous emergencies that are life-threatening might present in the newborn period of life. Thus, physicians need an in-depth understanding of such circumstances in order to treat critically ill neonates. Identification of these illnesses and choosing the appropriate course of action, which includes patient stabilization, differential diagnosis based on laboratory and imaging results, and well-guided therapy, are the responsibility of the emergency department staff. The most typical diagnoses in this kind of situation are severe bacterial infections, congenital heart illness, gastrointestinal crises (including malrotation with midgut volvulus, necrotizing enterocolitis, etc.), respiratory problems, neurologic abnormalities, and child abuse. Reviewing the most prevalent ailments of a severely unwell newborn in the emergency room is the major goal of this review article. In developing countries, neonatal mortality rates are a crucial determinant of their development. Management of emergencies especially in neonates can be very difficult and fatal if misdiagnosed. In this article, we will be discussing neonatal gastrointestinal (GI) emergencies.

## Introduction and background

**Figure 1:**
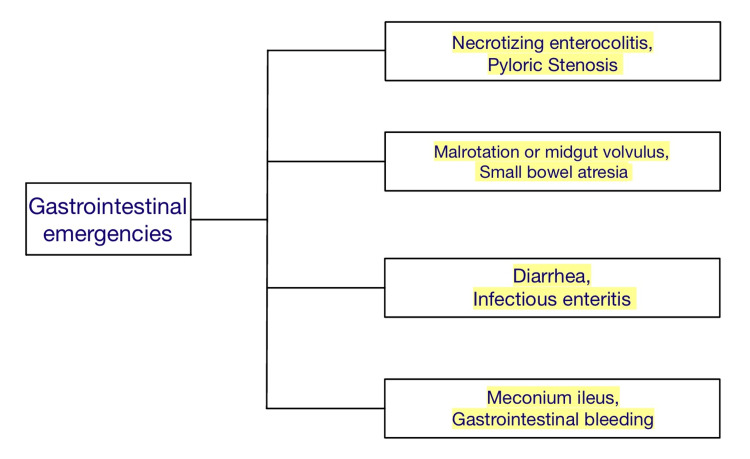
Shows gastrointestinal emergencies in neonates

Neonatal emergencies play a major role in health care, especially in a country like India, where there are not many health care facilities available, especially in the village areas of the country. Neonatal emergency manifest either at the moment of delivery, during the postpartum period in the hospital, or shortly after discharge at home. In many instances, emergencies can provide the practitioner with significant diagnostic and therapeutic challenges, thus they must be handled seriously. Early identification and treatment of infection, respiratory failure, shock, and other fetal illness like gastrointestinal (GI) emergencies in newborns (Figure 1) can significantly improve survival [1]. It is very crucial to identify the underlying causes and give established treatment for them. In a developing country like India, it is very important to decrease neonatal morbidity and mortality for healthy and safe lives as well as the development of the nation [2].

## Review

Necrotizing enterocolitis

Necrotizing enterocolitis (NEC) is a devastating disease of the newborn that presents in both pre-term and full-term neonates. NEC represents the most common neonatal GI emergency and is the commonest cause of GI perforation in this age group. Mortality related to this disorder is 10% to 50% [2]. Low birth weight and prematurity are risk factors for the development of NEC. Approximately 90% of cases are seen in premature neonates [3]. The true cause of NEC is unknown but is believed to be related to bowel wall injury, or hypoxia, which leads to inflammation and colonization of the bowel wall by bacterial flora. Some variation in the day of onset of the disease is possible and seems to be related to the gestational age and birth weight of the patient, thus, premature neonates present later with this illness than term infants [2-5].

One of the earliest symptoms of necrotizing enterocolitis is abdominal distention, which can be the presenting complaint by parents of neonates when they present in the emergency department (ED). The child may present with fever, irritability, lethargy, and peritoneal signs. As the course progresses, the child will begin to show clinical signs of shock, including hypotension, tachycardia, and tachypnea. Coffee ground or bilious emesis, guaiac positive, or grossly bloody stool may also develop at the time of evaluation. Shock indicates late presentation in cases of necrotizing enterocolitis and denotes an increased likelihood of intestinal perforation in such cases [3-5].

Diagnosis

Abdominal radiographs are the diagnostic modality of most importance in necrotizing enterocolitis cases. Pneumatosis intestinalis, portal vein air, and peritoneal air may all be seen on plain radiographs in many cases. Other radiographic signs that may be present earlier in the course include findings of bowel wall thickening and dilated loops of the bowel. Ultrasound evaluation can also reveal portal gas and ascites in some cases [2-5].

Management

Radiographic confirmation of the diagnosis necessitates prompt pediatric surgical consultation. Broad-spectrum antibiotics with coverage for intestinal bacteria should be started, along with fluid resuscitation. The patient is kept nil by mouth. A nasogastric tube should be inserted to decompress the stomach. Because of the severity of this condition, attention should be paid to the patient’s response to treatment, and plans for pediatric intensive care unit (PICU) placement should be initiated if needed [3-5].

Pyloric stenosis

Hypertrophic pyloric stenosis affects newborns between the ages of two to eight weeks and is a common reason for non-bilious vomiting. It is seen more commonly in male newborns. The diagnosis should be considered in a previously healthy child who develops non-bilious vomiting after the second week and who feeds vigorously after each episode of vomiting [6,7]. Hypertrophy of the pylorus is thought to occur due to abnormal development of the myenteric plexus of the pylorus. This leads to a lack of response to vasoactive neurotransmitters and hypertrophy of the pylorus. The resulting gastric outlet obstruction can lead to significant dehydration and metabolic derangements and requires prompt evaluation for surgical correction [6,7]. The child is typically a vigorous feeder and will initially attempt to feed after episodes of emesis. If the infant develops gastritis, there may be a history of coffee-ground emesis. A history of loose stools may lead to the misdiagnosis of gastroenteritis. Therefore, the diagnosis of pyloric stenosis must be considered in all infants between two and eight weeks of age, who present with significant non-bilious vomiting. Classically, a palpable “olive” in the epigastrium has been considered the physical exam finding pathognomonic of this disorder. Palpation of the hypertrophic pylorus requires a relaxed infant with an empty stomach. Peristaltic waves in the left upper quadrant may also be noted. Other examination findings of note are signs of dehydration and irritability in many cases [6,7].

Diagnosis and Management

Standard plain radiographs of the abdomen may show a distended stomach with or without peristaltic waves, the so-called caterpillar sign. The classic metabolic finding in an infant with pyloric stenosis is metabolic alkalosis with hypokalemia and hypochloremia, secondary to prolonged vomiting and loss of stomach acid. These findings may be absent if the infant presents early in the course of the disease [6,7]. Initial fluid resuscitation with normal saline (NS) should be followed by D5 0.45 NS (5% Dextrose and 0.45% Sodium Chloride) at 1.5 times the maintenance dosage to gradually correct the metabolic alkalosis. Potassium replacement with 10 to 20 mEq/L should be given if necessary, but only after urine output has been established. Placement of a nasogastric tube is not required and may cause an exacerbation of metabolic alkalosis secondary to continued loss of stomach acid. A pediatric surgeon should be consulted right away on identifying gastric outlet obstruction [6,7].

Malrotation or midgut volvulus

Malrotation refers to the failure of the embryonic gut to rotate two hundred and seventy degrees around the superior mesenteric vascular axis, resulting in a lack of the usual anatomic arrangement and stability of the small intestine in newborns. The distal duodenum does not arrive in the left abdomen and therefore, lacks the stability usually provided by the ligament of Treitz. The intestine is then predisposed to twisting around its single mesenteric attachment and obstructing the distal duodenum or proximal jejunum. This obstruction (or volvulus) may also be associated with vascular compromise secondary to twisting of the superior mesenteric artery and vein [8,9]. Ladd bands, which are fibrous attachments arising from the right peritoneal gutter may lead to intestinal obstruction. Midgut volvulus can cause ischemia and can cause loss of the entire midgut in a matter of hours and, therefore, represents a pediatric surgical emergency [8,9].

*Clinical Feature* 

In 80 to 100% of instances, these newborns present with bilious vomiting [8,9]. In children with midgut volvulus, bilious vomiting may be followed by abdominal distension, irritability, and blood in the child’s stool. Bowel necrosis may occur within two to four hours and shock can be seen in many cases. Physical examination may reveal progressive abdominal distension, peritoneal signs, guaiac-positive stool, or gross blood and signs of shock as the intestine becomes necrotic [8,9]. In a neonate with midgut volvulus, there will be a corkscrew sign seen on a plain radiograph, which denotes the jejunum twisting, and contrast will not cross over into the left side of the gut [9].

Management

Early consultation with a pediatric surgeon is essential. Resuscitation with normal saline and broad-spectrum antibiotics should be initiated if signs of perforation or shock are present. Due to the severity of this condition, plans for postoperative intensive care should be considered [10].

Small bowel atresia

Duodenal atresia is commonly associated with chromosomal abnormalities [11,12]. It is seen mostly in low birth weight or premature babies. Abdominal distension and vomiting are usually noted at the time of evaluation. Vomit is bilious in these patients and some may also present with jaundice. There will be a history of failure to pass meconium in newborns in the first 24 hrs. The infant may also present with tachycardia and signs of dehydration with decreased urine output [11,12].

Management

Supportive measures, including resuscitation with normal saline, should be initiated in response to signs of dehydration. If proximal small bowel obstruction is identified, a nasogastric tube for decompression may be indicated. Pediatric surgical consultation and admission of patients are necessary as operative management is almost always indicated [12].

Diarrhea

Diarrhea in the neonatal period can be very common, but if severe, can lead to life-threatening conditions. Aggressive management is sometimes needed to avoid severe dehydration, which is the main cause of neonatal mortality due to diarrhea. Diarrhea is caused by infection and inflammation of the GI system. Inflammation of the bowel wall can be caused by atopy, infection, and even ischemia. Other causes of diarrhea include congenital atrophy of the villi and short gut syndrome [13]. A complete history and physical examination are important. The amount of oral intake, urinary output, frequency and character of stools (whether bloody or non-bloody, as well as their color and consistency), change in behavior, irritability, and fever or other signs of infection must all be reviewed. Exposure to sick contacts (especially those with diarrheal illness) must be asked in the history of the patient. The presence of other children in the home and child care history should also be reviewed. Diarrhea is usually mild and self-limiting, but if severe, admission to the hospital and aggressive fluid therapy is essential [13,14]. Diarrhea will be further covered in the infectious enteritis topic of this article.

Infectious enteritis

Pathogens causing injury to the proximal small intestine lead to inflammation and dysfunction of the mucosa, producing watery diarrhea and sometimes vomiting, usually associated with viral pathogens. Bacterial pathogens usually affect the colon and blood may also be present in the stool. Rotavirus is the most common viral pathogen causing diarrhea and is most commonly seen in the winter season. It is associated with electrolyte abnormalities (including hypocalcemia), seizures, necrotizing enterocolitis, and an increased frequency of both apnea and bradycardia. Adenovirus, enteroviruses, and Norwalk agents, among other viral pathogens, also cause a similar presentation [15,16]. Escherichia coli (E. coli) has a number of forms and may have a variable presentation depending on the strain of the pathogen responsible for the infection. Enteroinvasive and enterohemorrhagic strains of E. coli present with bloody diarrhea, whereas other strains typically present with green watery diarrhea without blood or mucus. Other pathogens responsible for bloody diarrhea include Shigella, Campylobacter, Yersinia, and Aeromonas. Temperature and pulse are important indicators of infection and dehydration. A rectal examination is important for obtaining a stool sample but also for checking for anal fissures, as a possible etiology of bloody diarrhea. The patient should be weighed unclothed for an accurate weight. Close attention to respirations, detection of sunken fontanel, skin elasticity, whether a patient makes tears, mucous membranes, urinary output, and capillary refilling time will help determine the severity of dehydration [15,16].

Treatment

Mild dehydration (3% to 5%) should be treated with oral rehydration therapy if possible. If dehydration is moderate (6% to 9%) or severe (>10%), then generally IV fluid rehydration is needed. Antimotility agents such as loperamide, opium, and diphenoxylate should not be used. In addition, antibiotics should not be given unless culture results yield a specific bacterial agent. Antibiotics may disturb the patient's normal gut flora if misused. Noninvasive strains of E. coli can be treated with oral neomycin or colistin sulfate. Neomycin therapy may eradicate the bacteria and shorten the course of illness. An absorbable antibiotic, such as ampicillin or trimethoprim-sulfamethoxazole may be indicated for enteroinvasive bacteria causing diarrhea. Salmonella infection in the neonate can be treated with ampicillin, amoxicillin, Bactrim, cefotaxime, or ceftriaxone. Always consider admitting severely dehydrated neonates, as it can lead to mortality in many cases [15,16].

Meconium ileus

One of cystic fibrosis's early symptoms is meconium ileus. Meconium can create an intestinal obstruction in neonates. The blockage is usually located at the terminal ileum and, as such, causes a clinical picture consistent with distal small bowel obstruction. Ten percent to fifteen percent of children with cystic fibrosis will develop meconium ileus. In some cases, low birth-weight infants can also have meconium ileus [17,18]. When meconium fails to pass within 24 to 48 hours in a full-term infant, other conditions should also be considered. Ninety percent to ninety five percent of neonates with meconium ileus have a positive sweat chloride test [17]. Other GI manifestations of cystic fibrosis include constipation, distal intestinal obstructive syndrome, acquired megacolon, rectal prolapse, and pancreatitis. Meconium ileus is usually diagnosed prior to the postpartum discharge of the patient from the hospital when the child does not pass meconium in the first 24 to 48 hours of life. Bilious vomiting and abdominal distension are seen in these patients as meconium causes obstruction in the GI tract. The child may return to the ED after having treatment to relieve the obstruction, as infants with cystic fibrosis are prone to recurrence of intestinal obstruction. The abdomen will appear distended and tender. Loops of distended bowel may be palpable in some cases [17-19].

Management

Fortunately, the response to medical therapy is usually good. Enemas are largely effective, and contrast media may serve a therapeutic and diagnostic role in this regard. Correct dehydration and electrolyte imbalance in the patient, if present. If the obstruction cannot be alleviated after multiple enemas or if signs of perforation develop, surgical exploration is necessary. After relief from obstruction, recurrence should be prevented by proper treatment of the underlying cause. Intestinal malabsorption is typically treated with pancreatic enzyme preparation which helps in digestion [17-19].

GI bleeding

It can lead to serious, life-threatening conditions that require emergency surgical or medical interventions. A common cause of apparent GI bleeding is simply the swallowing of maternal blood at the time of birth or even from the cracked nipples of the breastfeeding mother. Detailed history and a careful physical examination are crucial when evaluating a neonate with apparent GI bleeding. In cases of significant GI bleeding, diagnostic evaluation with the lab test, radiographs, and appropriate consultation are required to ensure timely diagnosis and treatment with an examination of the anal area for fissures [20]. Neonates born in the out-of-hospital setting may present with hematemesis as a presenting sign of hemorrhagic disease of the newborn secondary to vitamin K deficiency [20,21]. Unwell neonates may also present with disseminated intravascular coagulation (DIC) or liver failure. Surgical emergencies such as necrotizing enterocolitis and midgut volvulus must also be considered. An anal fissure is the most common cause of rectal bleeding in the neonate. If the child is not septic, with no surgical emergency identified, and coagulation testing is normal, admission to the pediatrics service or gastroenterology unit is still appropriate in cases where alkali denaturation (Apt) test is positive and no rectal fissure or other benign explanation can be identified. The neonate may require endoscopy and both gastroenterology and pediatric surgeon consultation at the institution where the child is to be admitted. Maintaining homeostasis, identifying underlying causes, and treating the causes are the main aim of treatment in such a case [20,21].

## Conclusions

Although the presentations of many critical newborn illnesses might differ, most of them begin with no clear symptoms. From looking at the clinical results from several diverse causes of GI emergencies in newborns, it is particularly challenging to diagnose the emergency in neonates. Therefore, reliable radiological imaging is crucial for a prompt and correct diagnosis. For many conditions, plain abdominal radiography remains the gold standard. All medical professionals should be conversant with the fundamental radiological findings that could point to a gastrointestinal emergency. The infant mortality rate determines the developmental status and health care system of a country. So it is important for a physician to diagnose such gastrointestinal emergencies and treat them effectively in order to decrease neonatal morbidity and mortality in the community. In cases of GI bleeding, a local anal examination must be done to rule out cases of anal fissures. Misuse of antibiotics must be prevented as it can lead to damage to the normal gut flora of neonates. After arriving at the emergency room, a quick and accurate diagnosis should be established since neonatal cases can be serious. Early diagnosis can be achieved by an improved approach toward the patient and awareness of specific symptoms and radiological signs of the illness. 
